# Diagnostic utility of cerebrospinal fluid IgG index, 24-hour IgG synthesis rate, and immunophenotyping in primary central nervous system large B-cell lymphoma: A case report

**DOI:** 10.1097/MD.0000000000047208

**Published:** 2026-01-23

**Authors:** Yiqi Yan, Jing He, Jianguo Wu, Bihua Yao

**Affiliations:** a Department of Laboratory Medicine, The First People’s Hospital of Jiashan, Jiashan Hospital Affiliated to Jiaxing University, Jiaxing, Zhejiang, China; b Department of Clinical Laboratory, Zhejiang Provincial People’s Hospital, People’s Hospital of Hangzhou Medical College, Hangzhou, Zhejiang, China; c Department of Laboratory Medicine, Hangzhou TCM Hospital Affiliated to Zhejiang Chinese Medical University, Hangzhou, Zhejiang, China.

**Keywords:** case report, cerebrospinal fluid, flow cytometry, immunoglobulin G, primary central nervous system lymphoma

## Abstract

**Rationale:**

Primary central nervous system (CNS) large B-cell lymphoma (PCNS-LBCL) is a rare and aggressive brain malignancy that is challenging to diagnose early. Conventional imaging methods, such as magnetic resonance imaging and PET-CT, may not provide conclusive results in the early stages. This case highlights the diagnostic utility of cerebrospinal fluid (CSF) IgG index, 24-hour IgG synthesis rate, and immunophenotyping, offering a reliable noninvasive approach to improve early diagnosis when imaging results are inconclusive.

**Patient concerns:**

A 57-year-old male presented with progressively worsening headache, altered consciousness, and cognitive impairment, which developed gradually over several weeks. These symptoms prompted further diagnostic evaluation to identify the underlying cause.

**Diagnoses:**

Magnetic resonance imaging revealed nonspecific abnormalities, while PET-CT showed increased metabolic activity in regions suspicious for malignancy. CSF analysis demonstrated an elevated IgG index and 24-hour IgG synthesis rate, indicating CNS immune activation. Flow cytometry revealed abnormal B-lymphocytes, confirming the diagnosis of PCNS-LBCL.

**Interventions:**

The patient received a combined treatment regimen of R-CHOP chemotherapy and intrathecal methotrexate (MTX), chosen due to its ability to penetrate the blood–brain barrier and directly target CNS lesions. This combined approach is essential for treating PCNS-LBCL and preventing further neurological damage.

**Outcomes:**

Following treatment, the patient showed significant clinical improvement, with normalization of CSF parameters and recovery of neurological function, including improved cognitive abilities and motor skills. At the 1-year follow-up, imaging studies showed no relapse, and flow cytometry confirmed the absence of abnormal B-lymphocytes.

**Lessons:**

This case demonstrates the potential of integrating CSF IgG index, 24-hour IgG synthesis rate, and immunophenotyping as reliable, noninvasive diagnostic tools for the early detection of PCNS-LBCL, especially when imaging findings are inconclusive. The approach may offer valuable insights for improving diagnostic accuracy in clinical practice.

**Highlights**

1. A novel auxiliary diagnostic method combining CSF IgG index, IgG synthesis rate, and immunophenotyping.
2. Provides effective diagnostic support when MRI and PET-CT results are inconclusive.
3. Offers a noninvasive alternative for patients unsuitable for invasive brain biopsy.
4. Enhances early detection and supports timely clinical decision-making for PCNS-LBCL.

## 1. Introduction

Primary central nervous system (CNS) large B-cell lymphoma (PCNS-LBCL) is a rare and aggressive brain malignancy that usually has atypical clinical presentations, thereby making early diagnosis challenging.^[1–4]^ It is a highly aggressive tumor, and early diagnosis for optimal management is a key factor that may influence outcomes.^[5–7]^ Magnetic resonance imaging (MRI) and positron emission tomography–computed tomography (PET-CT) are tools that are commonly used for the diagnosis of brain diseases.^[8]^ However, their sensitivity and specificity are often insufficient,^[9,10]^ particularly if the lesions are small or located in hard-to-reach areas.^[9,11]^ Techniques such as flow cytometry and cerebrospinal fluid (CSF) cytology are highly specific. Nevertheless, they have limitations in obtaining early diagnosis.^[12]^ Flow cytometry requires high cell counts, and it may be influenced by cell debris and nonspecific staining. Meanwhile, CSF cytology is based on high-quality samples and the expertise of experienced pathologists.

Traditional biopsy is the gold standard for cancer diagnosis.^[13]^ However, it is an invasive procedure with associated risks such as bleeding and neurological complications and is not suitable for all patients.^[14]^ The prognosis of patients who relapse is poor, and further research should be performed to identify diagnostic biomarkers, more effective and less neurotoxic treatments, strategies that can improve drug penetration into the CNS, and immunotherapy.^[13]^

To address these challenges, this study proposed an innovative multivariable diagnostic approach that combines cerebrospinal fluid (CSF) IgG index, 24-hour IgG synthesis rate, and immunophenotyping parameters. This method is a noninvasive and highly sensitive and specific early diagnostic tool that overcomes the limitations of traditional imaging techniques. Therefore, it can significantly improve the diagnostic accuracy for PCNS-LBCL. By integrating these biomarkers,^[15]^ we do not only enhance diagnostic precision but also deepen our understanding on the pathophysiological mechanisms of PCNS-LBCL.^[16]^ In patients who cannot undergo traditional biopsy or those who are at higher risks, this method offers significant clinical potential, thereby reducing the need for invasive procedures and improving early diagnosis and treatment outcomes.

This study aimed to explore the clinical value of CSF analysis in the early diagnosis of PCNS-LBCL, focusing on the diagnostic potential of IgG index, IgG synthesis rate, and immunophenotyping parameters. Moreover, the future clinical applications of this noninvasive method was investigated.

### 1.1. Ethical review and informed consent

This case report was approved by the Zhejiang Provincial Medical Ethics Committee (Approval No.: Zhejiang Medical Ethics 2023 Total No. 407) on November 28, 2023. Informed consent was waived as the case involved anonymized patient data, in accordance with institutional and international ethical guidelines. The report was prepared following the CARE case report guidelines. The corresponding ethical approval forms and the author declaration form are provided in Supplementary Materials 11–13, Supplemental Digital Content, https://links.lww.com/MD/R162: Supplementary File 11, Supplemental Digital Content, https://links.lww.com/MD/R162 (Original ethical approval form in Chinese), Supplementary File 12, Supplemental Digital Content, https://links.lww.com/MD/R162 (English translation of the ethical approval form), and Supplementary File 13, Supplemental Digital Content https://links.lww.com/MD/R162 (Author declarations form).

## 2. Case presentation

### 2.1. Clinical presentation and clinical findings

A 57-year-old male presented with progressively worsening headache, altered consciousness, and cognitive impairment, accompanied by fatigue and decline in executive function over several weeks, prompting further diagnostic evaluation. The patient has a family history of hypertension but no significant genetic disorders. He is a nonsmoker and consumes alcohol occasionally. Psychological assessment revealed mild anxiety related to his health concerns. The patient also has a history of hypertension and hyperlipidemia, managed with medication, providing partial control of blood pressure and cholesterol, with no significant complications from these conditions.

Physical examination revealed altered consciousness and cognitive impairment, with mild disorientation and executive function decline. Vital signs were stable, and no focal neurological deficits were noted. The patient exhibited fatigue and generalized weakness, but no signs of infection or other systemic abnormalities were observed.

### 2.2. Timeline

The patient first experienced worsening headache, altered consciousness, and cognitive impairment on October 20, 2023. Over the next few weeks, symptoms worsened, prompting further evaluation. CSF analysis on October 27, 2023 showed an elevated IgG index and high protein levels. PET-CT and MRI revealed hypermetabolic regions in the brain. Flow cytometry confirmed the diagnosis of PCNS-LBCL. Treatment with R-CHOP chemotherapy and intrathecal methotrexate (MTX) started on October 30, 2023.

At the 6-month follow-up on April 30, 2024, the patient showed significant improvement with recovery of cognitive and motor skills. No relapse was observed on imaging, and flow cytometry confirmed the absence of abnormal B-lymphocytes.

### 2.3. Assistant examinations

Various diagnostic methods were used to confirm the diagnosis of PCNS-LBCL, including MRI, PET-CT, cerebrospinal fluid (CSF) analysis, and flow cytometry.

#### 2.3.1. *CSF analysis routine*

CSF examination revealed an elevated IgG index and increased 24-hour IgG synthesis rate, indicating CNS immune activation, which is characteristic of PCNS-LBCL. These findings strongly support a CNS origin for the pathology.

Comprehensive laboratory findings from CSF analysis, including biochemistry, immunofixation electrophoresis, immunotyping, and oligoclonal band tests, are provided in Supplementary Files 1–10, Supplemental Digital Content, https://links.lww.com/MD/R162.

CSF analysis revealed marked immune abnormalities, characterized by an elevated IgG index and an increased 24-hour IgG synthesis rate, supporting the diagnosis of PCNS-LBCL. These results strongly suggest that the pathology originated from the CNS (Table 1).

**Table 1:** CSF analysis results indicating abnormal immune response consistent with PCNS-LBCL.

Examination item	Result	Normal range	Clinical significance
White blood cell count	40↑/μL	0–5/μL	Indicates an abnormal immune response in the cerebrospinal fluid
24-h CSF IgG synthesis rate	260.92↑ mg/24 h	−9.90 to −3.30 mg/24 h	Increased, suggesting abnormal immune response, possibly indicating a tumor
IgG index	0.75↑	<0.7	Elevated, supporting overactivation of the immune system
Flow cytometry	Abnormal B-lymphocyte population	–	Identifies abnormal B-lymphocyte population, supporting PCNS-LBCL diagnosis

CSF = cerebrospinal fluid, PCNS-LBCL = primary central nervous system large B-cell lymphoma.

#### 2.3.2. *Positron emission tomography–computed tomography*

**Figure 1.**
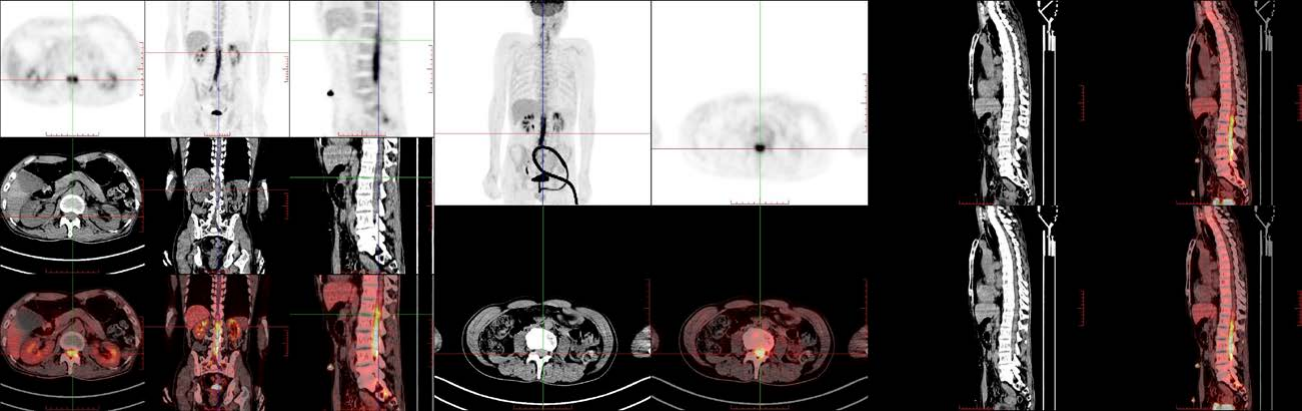
PET-CT image. The detection of hypermetabolic regions could provide indications for PCNS-LBCL and further emphasize the importance of cerebrospinal fluid analysis. PCNS-LBCL = primary central nervous system large B-cell lymphoma, PET-CT = positron emission tomography–computed tomography.

PET-CT (Fig. 1) revealed hypermetabolic regions in areas suspected to be malignant, which corresponded to clinical suspicions. These findings provided critical evidence when MRI results were inconclusive.

#### 2.3.3. Magnetic resonance imaging

**Figure 2.**
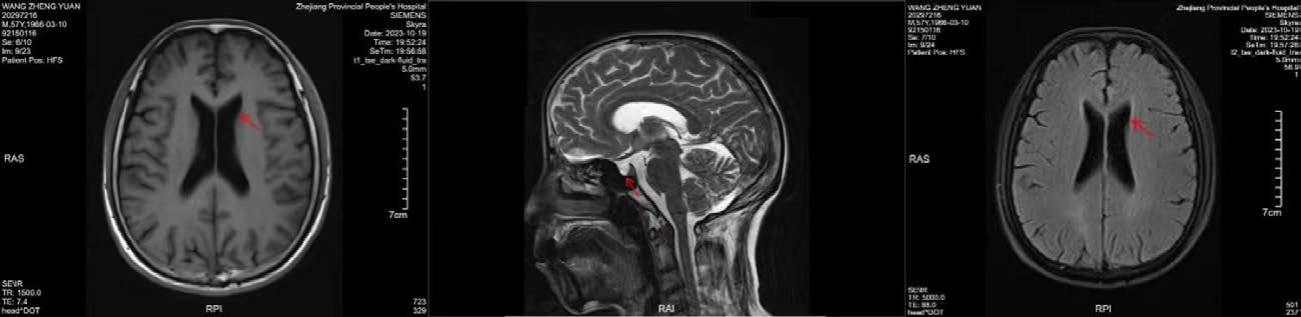
Contrast-enhanced head MRI showing a high signal in the white matter. With nonspecific abnormal signals on MRI, the possible locations of the lesions could be identified. Therefore, further analysis must be performed to validate the diagnosis. MRI = magnetic resonance imaging.

Brain MRI demonstrated nonspecific hyperintense signals within the frontal and parietal white matter (Fig. 2). Although these findings were consistent with the patient’s neurological symptoms, they were not sufficient to confirm PCNS-LBCL, necessitating further CSF-based analysis.

#### 2.3.4. Flow cytometry

Flow cytometry confirmed an abnormal B-cell population with κ light-chain restriction, λ light-chain deficiency, and high Ki-67 expression (93.43%), strongly indicating PCNS-LBCL.

**Figure 3.**
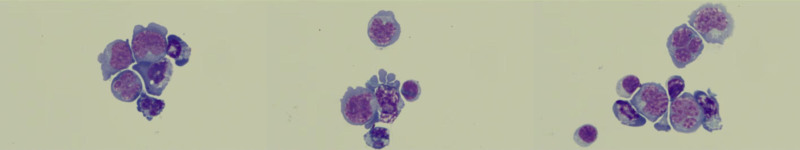
CSF cytology indicating 74% atypical lymphocytes. CSF = cerebrospinal fluid.

CSF cytology showed 74% atypical lymphocytes (Fig. 3), suggesting active B-cell proliferation. Flow cytometry immunophenotyping further confirmed the diagnosis by identifying abnormal B-lymphocytes with the aforementioned markers.

### 2.4. Summary

These diagnostic approaches, when used together, provided a comprehensive understanding of the disease. The combination of imaging (MRI and PET-CT) and CSF analysis confirmed the diagnosis of PCNS-LBCL. The integration of multiple diagnostic tools allowed for a more accurate and reliable diagnosis, overcoming the limitations of each method when used alone.

### 2.5. Final diagnosis

#### 2.5.1. Diagnostic methods CSF analysis

Elevated IgG index and 24-hour IgG synthesis rate, indicating CNS immune activation; MRI: Nonspecific white matter hyperintensities, suggestive of neurological involvement but not definitive; PET-CT: Revealed hypermetabolic regions in the brain, supporting CNS involvement; Flow cytometry: Identified abnormal B-cells with high Ki-67 expression, confirming the diagnosis.

#### 2.5.2. Diagnostic challenges

MRI findings were nonspecific, requiring additional tests (CSF analysis, PET-CT, and flow cytometry) for confirmation.

#### 2.5.3. Diagnosis

PCNS-LBCL was confirmed. Other diagnoses, such as multiple sclerosis and CNS infections, were considered but excluded.

#### 2.5.4. Prognosis

PCNS-LBCL carries a poor prognosis, particularly with CNS involvement. Early diagnosis and combined treatment with systemic chemotherapy and intrathecal MTX are essential for improving survival.

### 2.6. Interventions

The patient received R-CHOP chemotherapy (rituximab, cyclophosphamide, doxorubicin, vincristine, and prednisone) combined with intrathecal methotrexate (MTX). This regimen was chosen to target both systemic and CNS lesions, as R-CHOP alone may not adequately control CNS involvement in PCNS-LBCL.

- R-CHOP: Administered every 21 days for 6 cycles.
- Intrathecal MTX: Administered every 2 weeks to directly target CNS lesions.

There were no dose adjustments during treatment. The patient tolerated the regimen well, with mild side effects like nausea and fatigue, managed with supportive care. Although not widely recommended as first-line therapy, several studies have demonstrated its efficacy in high-risk patients.^[13,17,18]^

### 2.7. Outcomes and follow-up

**Clinician and patient-assessed outcomes** The patient improved clinically, with recovery of cognitive and motor skills, and normalization of CSF parameters.

**Important follow-up diagnostic and other test results** No relapse was seen on imaging, and flow cytometry showed no abnormal B-lymphocytes.

**Intervention adherence and tolerability** The patient followed the treatment plan and tolerated R-CHOP and intrathecal MTX well, with mild nausea.

**Adverse and unanticipated event**s No major adverse events occurred.

## 3. Discussion

### 3.1. Diagnostic value of combined CSF IgG index, IgG synthesis rate, and immunophenotyping

This study proposed a multivariable diagnostic approach that combined cerebrospinal fluid (CSF) IgG index, 24-hour IgG synthesis rate, and immunophenotypic analysis for the early diagnosis of PCNS-LBCL. Compared with traditional imaging methods (e.g., MRI and PET-CT), this approach could significantly enhance diagnostic sensitivity and specificity, particularly when imaging results are inconclusive. Hence, this method could be a noninvasive alternative for the early diagnosis of PCNS-LBCL. The core finding was that the combined use of CSF IgG index, IgG synthesis rate, and immunophenotyping parameters could improve early diagnostic accuracy. An elevated CSF IgG index (0.75) and 24-hour IgG synthesis rate (260.92 mg/24 hours) reflect abnormal immunoglobulin synthesis in the CNS, thereby suggesting early immunopathological changes in PCNS-LBCL. These biomarkers could help localize the disease source and reveal its immunological abnormalities. Immunophenotypic analysis, which detects cluster of differentiation10 + and cluster of differentiation20 + B-cell markers, further enhances diagnostic reliability, particularly when the imaging result is unclear. Thus, the integration of CSF IgG index and immunophenotyping parameters is a more precise tool for early diagnosis, particularly in cases with atypical imaging findings.

### 3.2. Comparison with conventional diagnostic methods and literature evidence

Stereotactic brain biopsy is the gold standard for diagnosing PCNS-LBCL. However, it cannot be widely used due to its invasiveness and associated risks.^[4,11]^ The multivariable diagnostic approach presented in this study, which integrates the CSF IgG index, IgG synthesis rate, and immunophenotyping, offers a noninvasive alternative, particularly for patients who are not candidates for biopsy. Previous studies have shown that CSF cytology and immunophenotyping have a high sensitivity for diagnosing PCNS-LBCL.^[19]^ By introducing the IgG index, this study further enhances diagnostic accuracy. MRI and PET-CT contribute to the diagnosis of PCNS-LBCL. However, they often lack sufficient specificity, particularly in the early stages when lesions are small or imaging findings are atypical.^[20]^ The proposed multivariable approach provides a more specific and sensitive diagnostic tool, particularly when imaging results are inconclusive, thereby offering significant advantages in early diagnosis. Hui Zhao et al^[19]^ showed that cerebrospinal fluid cytology has a high specificity in diagnosing PCNS-LBCL. we utilized approximately 74% of atypical lymphomas, based on elevated cerebrospinal fluid white blood cell counts combined with immunophenotyping (CD10+, CD20+, Ki-67+, B-cell lymphoma 2 (bcl-2)dim, κexpression, and λdeficiency), to diagnose Diffuse Large B-Cell Lymphoma (DLBCL). By incorporating the IgG index and 24-hour synthesis rate, the diagnostic approach for PCNS-LBCL was improved, with a better sensitivity and specificity than traditional MRI and PET-CT, which provides reliable early diagnostic evidence.

### 3.3. Quantitative thresholds and interpretation of CSF immunoglobulin indices

The CSF IgG index is an important auxiliary diagnostic marker for CNS diseases. SAADEH et al^[21]^ showed that the IgG index and 24-hour IgG synthesis rate can effectively distinguish inflammatory from non-inflammatory lesions. Saadeh et al further provided quantitative reference thresholds for CSF immunoglobulin parameters. In their Mayo Clinic cohort, an IgG index >0.7 indicated intrathecal IgG synthesis with 92.1 % specificity and 49.7 % sensitivity, while a 24-hour IgG synthesis rate >12 mg/24 hours showed 92.4 % specificity and 21.9 % sensitivity. These cutoffs have been widely adopted to distinguish abnormal intrathecal antibody production from passive diffusion across the blood–brain barrier. In the present case, both parameters were markedly elevated (IgG index = 0.75; IgG synthesis rate = 260.92 mg/24 hours), far exceeding these thresholds. When integrated with immunophenotyping findings of κ-restricted CD20+/CD10 + B-cell proliferation, these quantitative indicators provide an evidence-based framework for confidently differentiating PCNS-LBCL from other CNS inflammatory disorders.

### 3.4. Supporting evidence from neurological and immunophenotypic studies

Murakami et al^[22]^ found that the IgG index is also closely related to the pathophysiology of Lewy bodies in Parkinson disease. In addition, Gadoth et al^[23]^ revealed that elevated LGI1-IgG CSF indices are associated with more severe neurological damage. These studies validate the diagnostic value of IgG indices in neurological diseases. Further, they support the application of these indices in the early diagnosis of PCNS-LBCL in this study. Immunophenotypic analysis plays an important role in the diagnosis of brain tumors. Mitrofanova et al^[24]^ have shown that different immunophenotypic markers (such as CD117/CD34/connexin43/NeuroD1) can be used to distinguish different cell types in gliomas. Our findings showed that the combination of the IgG index, IgG synthesis rate, and immunophenotypic analysis can significantly improve the accuracy of early diagnosis, thereby offering novel prospectives for the development of noninvasive diagnostic tools.

### 3.5. Strengths and limitations of the proposed diagnostic approach

The strength of this study is that it presented a noninvasive tool. By combining CSF analysis, immunophenotyping, and the IgG index, it enables accurate early diagnosis, particularly for patients who cannot undergo biopsy. Further, it overcomes the limitations of using single markers. However, the method presented in this research has certain limitations. An elevated IgG index and IgG synthesis rate were not specific to PCNS-LBCL. This is because similar increases can also be observed in other CNS disorders, such as multiple sclerosis, neurosyphilis, and acute inflammatory demyelinating polyneuropathy.^[25–31]^ However, these conditions can be effectively differentiated when combined with clinical symptoms and other diagnostic tests. For example, Guillain–Barré syndrome is characterized by acute muscle weakness and cerebrospinal fluid albuminocytologic dissociation. Other tumors, such as non-LBCL-type primary CNS lymphoma and metastatic brain tumors,^[32]^ have distinct radiological features. Neurosyphilis is characterized by elevated CSF protein levels and lymphocytic pleocytosis, with serological testing useful for identifying it.^[28,29]^ In this study, the combined use of an elevated IgG index and increased synthesis rate and the identification of abnormal B-cell populations via flow cytometry can effectively differentiate PCNS-LBCL from other diseases.

### 3.6. Clinical implications and future perspectives

This study presented a novel, noninvasive diagnostic approach by combining CSF IgG index, IgG synthesis rate, and immunophenotyping, thereby making it particularly suitable for patients who cannot undergo biopsy. In patients with PCNS-LBCL, early diagnosis not only provides an opportunity for timely treatment but also reduces the need for invasive procedures such as biopsies, thereby decreasing treatment risks. The multivariable method proposed in this study, which integrates immunological and molecular markers, offers a more accurate early diagnosis. This method is valuable when imaging results are inconclusive, providing a basis for early intervention and treatment, such as the initiation of R-CHOP combined with intrathecal methotrexate chemotherapy,^[17]^ which significantly improves patient prognosis.

The multivariable diagnostic approach in this study had good sensitivity and specificity for the early diagnosis of PCNS-LBCL. However, it had some limitations. The specificity of the CSF IgG index and synthesis rate is low, which may lead to misdiagnosis or overdiagnosis. In addition, the sample size was small. In particular, only 1 case was included, which could have limited statistical significance. Therefore, multicenter, large-sample clinical trials should be performed to increase the sample size and validate the generalizability of this method. To enhance diagnostic accuracy, incorporating molecular markers such as circulating tumor DNA or proteomics is recommended. Moreover, due to the heterogeneity of PCNS-LBCL, integrating genomic and transcriptomic features could further refine the diagnostic model. Long-term follow-up studies can help evaluate the impact of this method on clinical outcomes.

**Figure 4.**
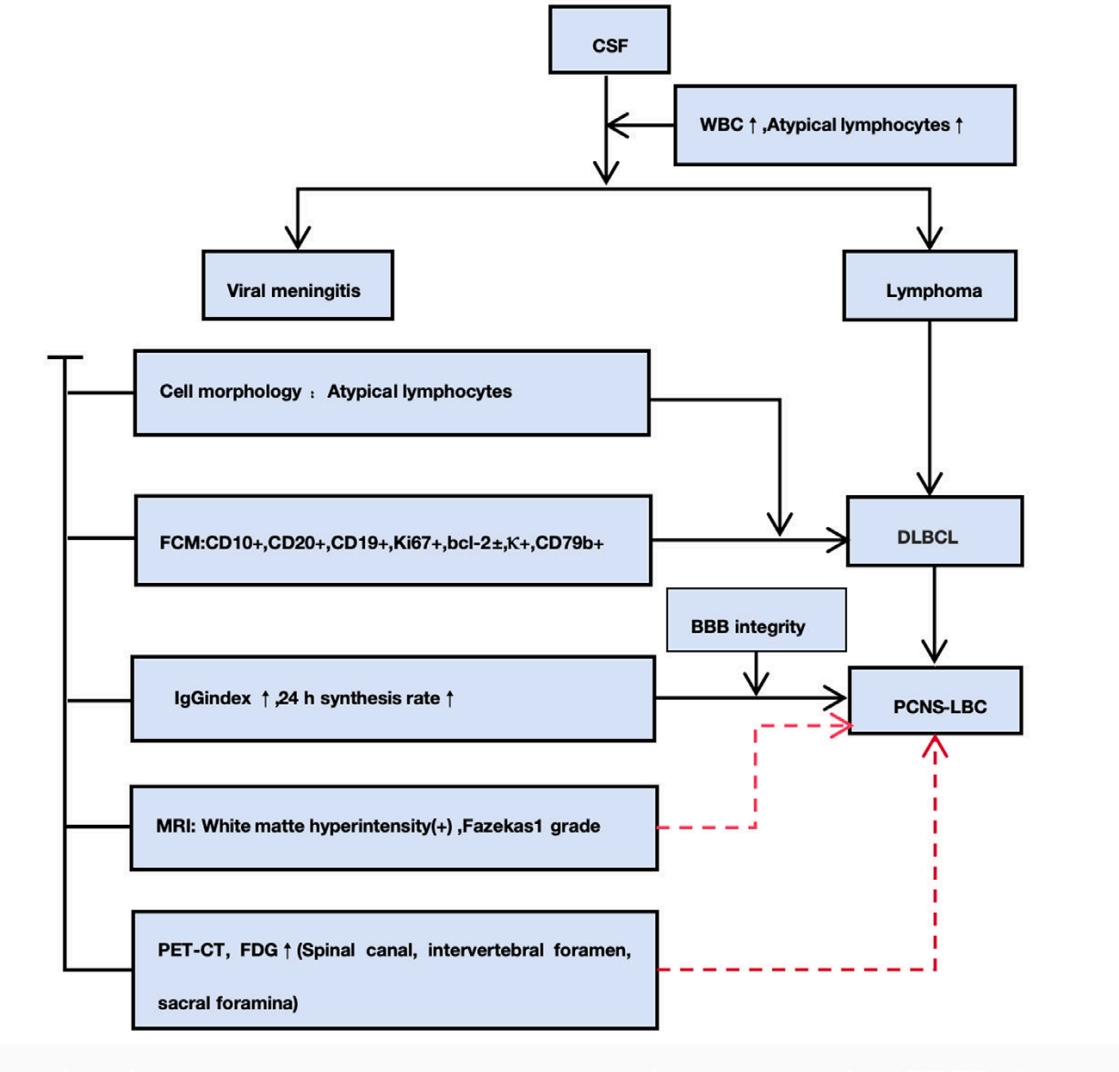
Diagnostic flowchart of PCNS-LBCL based on the CSF examination. CSF = cerebrospinal fluid, PCNS-LBCL = primary central nervous system large B-cell lymphoma.

Finally, the novel indexes proposed in this paper, which incorporates the IgG index and 24-hour synthesis rate, was used to obtain the diagnosis of PCNS-LBCL (Fig. 4).

## 4. Patient perspective

The patient expressed relief at avoiding an invasive biopsy. While he experienced mild side effects, including nausea, he reported improvement in cognitive and motor functions following treatment, which positively impacted his overall well-being.

## 5. Conclusions

This case report proposes a noninvasive auxiliary diagnostic approach combining CSF IgG index, 24-hour IgG synthesis rate, and immunophenotyping analysis. This method reduces the reliance on invasive procedures such as brain biopsy and provides critical support for preoperative decision-making by neurosurgeons. Future studies are needed to validate its broader applicability in CNS diseases.

## Acknowledgments

The authors want to thank the Youth members in the Department of Clinical Laboratory, Zhejiang Provincial People’s Hospital, for providing encouragement and support. We thank Bullet Edits Limited for the linguistic editing and proofreading of the manuscript. All individuals listed in the acknowledgments section have given their permission to be named. A summary of the study highlights is provided in Supplementary File 14 https://links.lww.com/MD/R162.

## Author contributions

**Conceptualization:** Yiqi Yan, Jianguo Wu.

**Data curation:** Yiqi Yan, Jianguo Wu.

**Formal analysis:** Bihua Yao, Yiqi Yan.

**Funding acquisition:** Bihua Yao, Jianguo Wu.

**Investigation:** Jing He, Jianguo Wu.

**Methodology:** Yiqi Yan, Jing He.

**Project administration:** Jing He, Bihua Yao.

**Resources:** Jing He, Bihua Yao.

**Software:** Bihua Yao.

**Writing – original draft:** Yiqi Yan, Bihua Yao.

**Writing – review & editing:** Jianguo Wu, Bihua Yao.

## Supplementary Material


