# Supplementary material for: Diagnostic utility of cerebrospinal fluid IgG index, 24-hour IgG synthesis rate, and immunophenotyping in primary central nervous system large B-cell lymphoma: A case report
Source: Medicine (Baltimore). 2026 Jan 23;105(4):e47208. doi: 10.1097/MD.0000000000047208 (PMC12851708; doi:10.1097/MD.0000000000047208)
Supplement: Supplementary file 1 [file medi-105-e47208-s001.pdf]

# 浙江省人民医院检验中心检验报告单



| | | | |
|---|---|---|---|
| 姓　　名: **王正源** | 病 员 号: **92150116** | 标本种类: 脑脊液 | 样本编号: 20231027G0140202 |
| 类　　别: **住院** | 床　　号: 25 | 标本性状: | 条型码号: 014381939200 |
| 科　　别: **血液病科** | 性　　别: 男 | 送检医生: 00001845_陈一瑞 | 采集时间: |
| 病　　区: **1-20病区** | 年　　龄: 57岁 | 接收人员: 陆驾文 | 接收时间: 2023-10-27 11:40 |
| 送检单位: | 采集部位: 脊柱 | 临床初诊: 颈痛 | |
| 备　　注: | | | |

| NO | 项目 | 结果 | 参考区间 | 单位 | 实验方法 |
|---|---|---|---|---|---|
| 1 | 颜色 | 无色 | 无色 | | 目测法 |
| 2 | 透明度 | 透明 | 透明 | | 目测法 |
| 3 | 有核细胞计数 | 40.0 | | 个/μl | 显微镜法 |
| 4 | 体液淋巴细胞分类 | 15.0 | | % | 显微镜法 |
| 5 | 体液中性细胞分类 | 4.0 | | % | 显微镜法 |
| 6 | 体液巨噬细胞分类 | 6.0 | | % | 显微镜法 |
| 7 | 体液红细胞计数 | 1.0 | | 个/μl | 显微镜法 |
| 8 | 潘氏试验 | 阳性(+) ↑ | | | 饱和石炭酸法 |
| 9 | 成熟浆细胞 | 1 | ≤2 | 个 | 显微镜法 |
| 10 | 异常细胞 | 74.00 | | % | 显微镜法 |

**检查结果:**

涂片有核细胞数量中等，以异常淋巴细胞为主，该类细胞胞体偏大不规则，胞浆丰富着色深蓝，胞核较大，染色质疏松，着色深紫色，核仁明显，1~2个。可见少量成熟淋巴细胞及巨噬细胞，偶见中性粒细胞，未见细菌、真菌及其他特殊异常细胞。

**诊断及建议、评估:**

涂片以异常淋巴细胞为主，考虑淋巴瘤细胞，请结合临床。

**图像:**

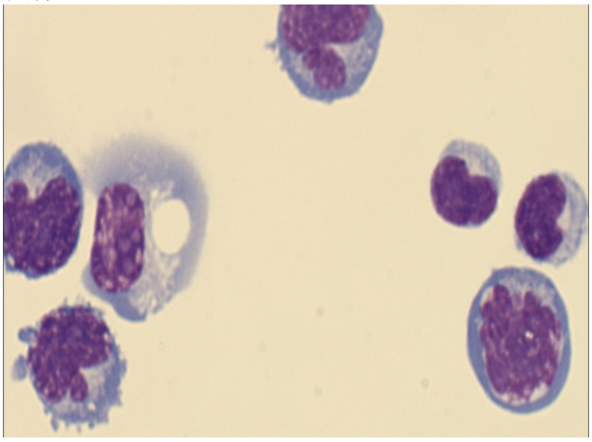
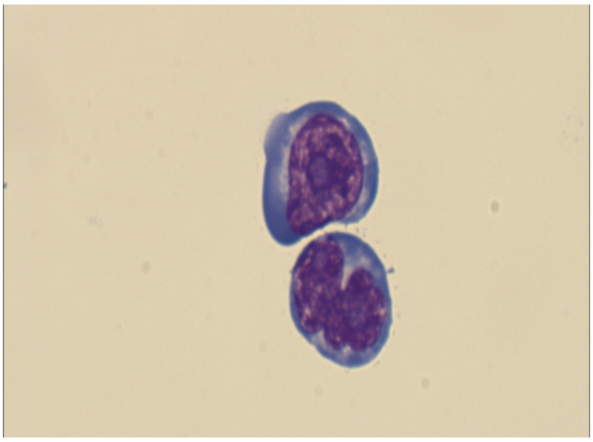
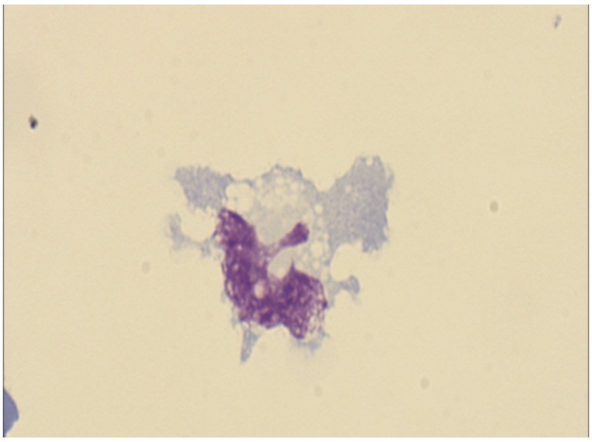
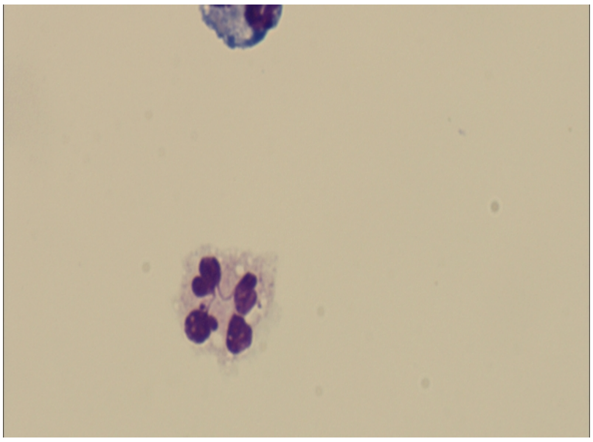

报告时间: 2023-10-27 16:07　　检验:　　审核:

注: 此检验报告仅对本次标本负责。
如有疑问，请在一周内与检验中心细胞室联系，电话：0571-85893261。

**Test report from the Laboratory Center of Zhejiang Provincial Peoples Hospital**

**Name**: Wang Zhengyuan
**Category**: Inpatient
**Department**: Hematology
**Gender**: Male
**Ward**: 25 (Room 1, Section 20)
**Age**: 57 years old
**Submitting Unit**: Specimen Collection and Delivery Room
**Specimen Type**: Pleural Fluid
**Specimen Property**: One-Time Sample
**Specimen Number**: 014389139200
**Delivering Doctor**: 00001845, Chen Yi
**Receiving Personnel**: Chen Wen
**Clinical Diagnosis**: Pleural Effusion
**Collection Time**: 2023-10-27, 1

| No. | Item | Result | Reference Range | Unit | Test Method |
|---|---|---|---|---|---|
| 1 | Color | Colorless | Colorless | | Visual Method |
| 2 | Transparency | Transparent | Transparent | | Visual Method |
| 3 | Nucleated Cell Count | 40.0 | | ↑/μL | Microscopy |
| 4 | Lymphocyte Classification | 15.0 | % | % | Microscopy |
| 5 | Monocyte Classification | 46.0 | % | % | Microscopy |
| 6 | Neutrophil Classification | 4.0 | % | % | Microscopy |
| 7 | Eosinophil Classification | 1.0 | % | % | Microscopy |
| 8 | Red Blood Cell Count | 0 | ↑/μL | ↑/μL | Microscopy |
| 9 | Rivalta Test | Positive (+) | ↑ | | Chemical Precipitation |
| 10 | Atypical Cells | 74.00 | % | % | Microscopy |

## Examination Results:

The sample contains a moderate number of nucleated cells, predominantly atypical mesothelial cells. These cells show significant irregularities in morphology, with deeply stained and large nuclei, loose chromatin, prominent nucleoli, and a high nucleocytoplasmic ratio (>1:2). A small number of reactive mesothelial cells and macrophages were observed, along with occasional neutrophils. No fungi or other specific abnormal cells were identified.

**Diagnosis and Recommendations:**
The smear is predominantly composed of abnormal lymphocytes, suggestive of lymphoma cells. Please correlate with clinical findings.

**Images:**

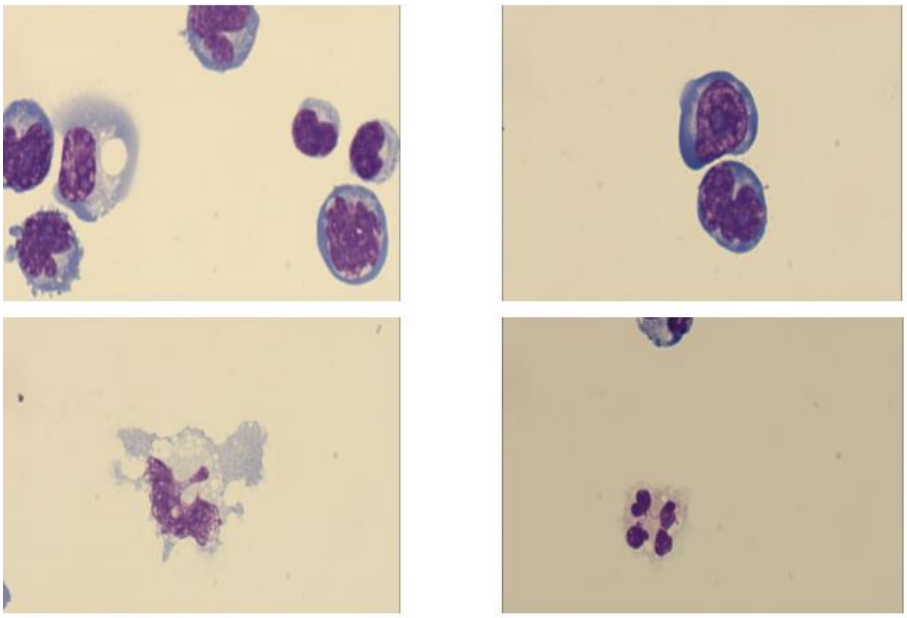

# 浙江省人民医院检验中心检验报告单



姓　　名: **王正源**　　病 员 号: **92150116**　　标本种类: 脑脊液　　样本编号: 20231027G0231039
类　　别: **住院**　　床　　号: 25　　标本性状:　　条型码号: 014381939301
科　　别: **血液病科**　　性　　别: 男　　送检医生: 00001845_陈一瑞　　采集时间:
病　　区: **1-20病区**　　年　　龄: 57岁　　接收人员: 陆驾文　　接收时间: 2023-10-27 11:39
送检单位:　　采集部位: 脊柱　　临床初诊: 颈痛
备　　注:

| NO | 项目 | 结果 | | 参考区间 | 单位 | 实验方法 |
|---|---|---|---|---|---|---|
| 1 | 脑脊液蛋白定量 | 570.3 | ↑ | ≤45.0 | mg/dl | 终点法 |
| 2 | 脑脊液糖定量 | 0.05 | ↓ | 2.50-4.50 | mmol/L | 己糖激酶法 |
| 3 | 脑脊液乳酸脱氢酶 | 331 | ↑ | 8-32 | U/L | 速率法 |
| 4 | 脑脊液氯化物 | 120.6 | | 120.0-132.0 | mmol/L | 离子选择性电极法 |
| 5 | 脑脊液乳酸 | 12.4 | ↑ | 1.1-2.4 | mmoL/L | 乳酸氧化酶法 |

报告时间:　2023-10-27 15:03　　检验: 张学童　　审核: 孙汝林

注: 此检验报告仅对本次标本负责。　"HR"标注为浙江省临床检验中心公布的互认检验项目
如有疑问，请在一周内与检验中心生化室联系，电话：0571-85893265。

**Zhejiang Provincial People's Hospital Laboratory Center Examination Report**
**Cerebrospinal Fluid Biochemistry**

Name: Wang Zhengyuan Patient No.: 92150116
Category: Inpatient Ward: 25
Department: Hematology Gender: Male
Room: 1-20 Ward Section Age: 57 years old
Submitting Unit: Specimen Collection Room
Specimen Type: Cerebrospinal Fluid
Specimen Nature: Routine Sample
Specimen Number: 00001845, Chen Yi
Receiving Personnel: Chen Wen
Clinical Diagnosis: Headache
Collection Time: 2023-10-27, 11:39

| No. | Item | Result | Reference Range | Unit | Test Method |
|---|---|---|---|---|---|
| 1 | CSF Protein Level | 570.3 ↑ | <45.0 | mg/dl | Turbidimetry |
| 2 | CSF Glucose Level | 0.05 ↓ | 2.50 - 4.50 | mmol/L | Enzymatic Method |
| 3 | CSF Lactate Dehydrogenase (LDH) | 331 ↑ | 8 - 32 | U/L | Rate Method |
| 4 | CSF Chloride | 120.6 | 120.0 - 132.0 | mmol/L | Ion-Selective Electrode Method |
| 5 | CSF Lactate | 12.4 ↑ | 1.1 - 2.4 | mmol/L | Lactic Acid Oxidase Method |

# 浙江省人民医院检验中心检验报告单



姓　　名: **王正源**　病 员 号: **92150116**　标本种类: 血清　样本编号: 20231024G0326001
类　　别: **住院**　床　　号: 53　标本性状:　条型码号: 014371359900
科　　别: **神经内科**　性　　别: 男　送检医生: 00001234_王奕琪　采集时间: 2023-10-20 15:58
病　　区: **5-10病区**　年　　龄: 57岁　接收人员: 5315|杨政权　接收时间: 2023-10-20 17:08
送检单位:　采集部位: 静脉　临床初诊: 颈痛
备　　注:

| NO | 项目 | 结果 | 参考区间 | 单位 | 实验方法 |
|---|---|---|---|---|---|
| 1 | 免疫固定分型IgG | 阴性(-) | 阴性(-) | | 免疫固定电泳 |
| 2 | 免疫固定分型IgA | 阴性(-) | 阴性(-) | | 免疫固定电泳 |
| 3 | 免疫固定分型IgM | 阴性(-) | 阴性(-) | | 免疫固定电泳 |
| 4 | 免疫固定分型κ链 | 阴性(-) | 阴性(-) | | 免疫固定电泳 |
| 5 | 免疫固定分型λ链 | 阴性(-) | 阴性(-) | | 免疫固定电泳 |

报告时间:　2023-10-24 10:32　检验: 陈[illegible]　审核: [illegible]

注: 此检验报告仅对本次标本负责。
如有疑问，请在一周内与检验中心免疫室联系，电话：0571-85893262。

**Zhejiang Provincial People's Hospital Laboratory Center Examination Report**

**Immunofixation Electrophoresis**

**ame: Wang Zhengyuan  Patient No.: 92150116**
**Category: Inpatient Ward: 53**
**Department: Neurology Gender: Male**
**Room: 5–10 Ward Section  Age: 57 years old**
**Submitting Unit: Quiet Vein Collection Room**
**Specimen Type: Blood Serum**
**Specimen Property: Routine Sample**
**Submitting Doctor: 00001234, Wang Xueqi**
**Receiving Personnel: 5315, Yang Zhengquan**
**Clinical Diagnosis: Headache**
**Collection Time: 2023-10-20, 15:58**
**Receiving Time: 2023-10-20, 17:08**

| No. | Item | Result | Reference Range | Unit | Test Method |
|---|---|---|---|---|---|
| 1 | Immunofixation Typing IgG | Negative (-) | Negative (-) | | Immunofixation Electrophoresis |
| 2 | Immunofixation Typing IgA | Negative (-) | Negative (-) | | Immunofixation Electrophoresis |
| 3 | Immunofixation Typing IgM | Negative (-) | Negative (-) | | Immunofixation Electrophoresis |
| 4 | Immunofixation Typing κ Chain | Negative (-) | Negative (-) | | Immunofixation Electrophoresis |

# 浙江省人民医院临床实验室检测报告单



| | | | |
|---|---|---|---|
| 姓　　名: **王正源** | 病 员 号: **92150116** | 标本种类: 脑脊液 | 样本编号: 20231027G0960001 |
| 类　　别: **住院** | 床　　号: 25 | 标本性状: | 条型码号: 014381939100 |
| 科　　别: **血液病科** | 性　　别: 男 | 送检医生: 00001845_陈一瑞 | 采集时间: |
| 病　　区: **1-20病区** | 年　　龄: 57岁 | 接收人员: 倪万茂 | 接收时间: 2023-10-27 11:45 |
| 送检单位: | 采集部位: | 临床初诊: 颈痛 | |
| 备　　注: | | | |

**描 述:**

异常B淋巴细胞群约占总数的67.13%，CD19++、SSC大、FSC大、CD45++、CD33－、CD13－、CD117－、HLA－DR－、CD7－、CD16－、κ+、λ－、CD34－、CD38+、CD10+、CD20++、CD5－、CD200 dim、FMC7－、sIgM++、CD23－、CD138－、CD79b+、CD43+、CD22+、CD9－、CD103－、CCR6－、CD49d dim、bcl－2 dim、Ki67+（93.43）。

**结 果:** 本次脑脊液样本，异常B淋巴细胞群约占总数的67.13%，κ单型性表达、λ缺失、CD34-、CD38+、CD10+、CD20++、CD5-、bcl-2 dim、Ki67+（93.43%），与2023.10.23脑脊液表型一致，倾向于DLBCL。

报告时间:　2023-10-27 14:10　　检验: 王蕾　　审核: 朱和芳

注: 此检验报告仅对本次标本负责。
如有疑问，请在一周内与分子诊断实验室联系，电话：0571-85893550。

**Zhejiang Provincial People's Hospital Clinical Laboratory Testing Report**
**30-Item Tissue Immunophenotyping**

Name: Wang Zhengyuan Patient No.: 92150116
Category: Inpatient Ward: 25
Department: Hematology Gender: Male
Room: 1-20 Ward Section Age: 57 years old
Specimen Type: Cerebrospinal Fluid
Specimen Nature: Routine Sample
Specimen Number: 00001845, Chen Yi
Receiving Personnel: Chen Wen
Clinical Diagnosis: Headache
Collection Time: 2023-10-27, 11:40
Receiving Time: 2023-10-27, 11:45

**Description:**
Abnormal B lymphocyte population accounts for approximately 67.13%. These cells exhibit the following characteristics:
CD19++, SSC++, FSC++, CD45++, CD33-, CD13-, CD117-, HLA-DR-, CD7-, CD16-, κ+, λ-, CD34-, CD38++, CD10++, CD20++, CD5-, CD200 dim, FMC7-, IgM++, CD23-, CD138-, CD79b++, CD43+, CD22+, CD9-, CCR6-, CD49d dim, bcl-2 dim, Ki67+ (93.43%).

**Results:**
This cerebrospinal fluid sample shows an abnormal B lymphocyte population accounting for 67.13%. These cells are monoclonal in nature, characterized by CD34-, CD38++, CD10++, CD20++, CD5-, bcl-2 dim, and Ki67+ (93.43%). The findings from the cerebrospinal fluid immunophenotyping on 2023-10-23 are consistent, suggesting DLBCL (Diffuse Large B-Cell Lymphoma).

# 杭州迪安医学检验中心检测报告单

HangZhou DIAN Medical Laboratory Test Report

## 寡克隆区带综合分析报告

**送检单位：浙江省人民医院** **条码号：100622715574**

| | | | |
|---|---|---|---|
| 姓名：王正源 | 病人类别：住院 | 科别：5-10病区（神经内科） | 床号：53 |
| 性别：男 | 病人电话：13175252562 | 门诊/住院号：92150116 | 样本类型：脑脊液，血清 |
| 年龄：57岁 | 送检医生：王奕琪 | 临床诊断：颈痛 | 样本状态：外观正常 |

### 蛋白定量分析

| 项目名称 | 脑脊液（mg/L） | 血清（g/L） | ALB Index | ALB Index 参考范围 | IgG Index | IgG Index 参考范围 |
|---|---|---|---|---|---|---|
| ALB | 6060.0 | 47.6 | 127.31 ↑ | <9.00 | 0.75 ↑ | <0.70 |
| IgG | 1250.0 | 13.10 | | | | |

### 24h IgG合成率

| - | 结果（mg/24h） | 参考值（mg/24h） |
|---|---|---|
| Tourtellotte 公式 | 260.92 ↑ | -9.90～3.30 |

### 寡克隆区带电泳

- ☑ Ⅰ型. CSF和血清中均未见OB条带
- ☐ Ⅱ型. 仅于CSF中见到OB条带
- ☐ Ⅲ型. CSF中OB条带数＞血清
- ☐ Ⅳ型. CSF和血清中可见对称性OB条带
- ☐ Ⅴ型. CSF和血清中均检出单克隆条带

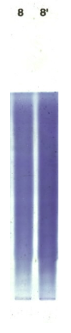


CSF S

寡克隆区带图谱

### 寡克隆区带电泳项目解释

| 分型 | 界定描述 | 提示 | 临床意义 |
|---|---|---|---|
| Ⅰ | 阴性 | 中枢神经系统（CNS）无鞘内IgG合成 | 见于健康人及其他非炎症性神经系统疾病（NIND）患者 |
| Ⅱ | 阳性 | 脑脊液电泳条带为中枢神经系统B细胞反应所致，提示CNS有鞘内IgG合成 | 主要见于多发性硬化（MS）患者；少部分见于其他CNS脱髓鞘病、自身免疫性脑炎、CNS感染、风湿神经病等 |
| Ⅲ | 阳性 | 血清与脑脊液相同的条带为系统性B细胞反应所致，仅见于脑脊液的条带则为中枢神经系统B细胞反应所致，提示CNS有鞘内合成且血脑屏障有破坏 | 主要见于继发性脱髓鞘疾病、脑膜癌和脑膜炎等；少部分MS患者OB结果亦可表现为此型 |
| Ⅳ | 阴性 | 血清与脑脊液呈镜像分布的寡克隆区带，提示病变在中枢神经系统之外，存在系统性B细胞反应且伴有血脑屏障破坏 | 主要见于格林巴利综合征，系统性自身免疫病等；偶见于MS以外的其他CNS脱髓鞘病、CNS感染等 |
| Ⅴ | 阴性 | 提示存在单克隆增殖的副蛋白 | 主要见于血液病（如淋巴瘤、多发性骨髓瘤等）继发神经系统并发症患者 |

注：脑脊液寡克隆区带阳性表示存在鞘内合成，是指Ⅱ型和Ⅲ型，有助于多发性硬化（MS）的诊断，但非MS所特有

### 结果小结

脑脊液白蛋白指数高于正常参考范围；脑脊液24h IgG合成率高于正常参考范围；CSF和血清中均未检出OB条带，请结合临床分析。

**此标本已核对复查。**

※本结果只对此条码来样负责，如有疑问，请在报告单发布一周内提出。



检验人员：韩晓娅 审核人员：彭义忠

采样时间：2023-10-20 15:58 接收时间：2023-10-20 20:31 检验时间：2023-10-20 22:16 报告时间：2023-10-22 16:50

杭州迪安医学检验中心有限公司 检验专用章

电子报告

**地址：杭州市西湖区三墩镇金蓬街329号1号楼** **电话：400 7118 000**

## Hangzhou DIAN Medical Laboratory Test Report
## Comprehensive Analysis Report of Barrier Region Bands

**Submitting Unit:** Zhejiang Provincial People's Hospital

**Name: Wang Zhengyuan    Category: Inpatient    Department: Neurology (5–10 Ward Section)**

**Gender: MaleAge: 57 years old  Patient No.: 92150116**

**Bed No.: 53    Test Type: Cerebrospinal Fluid, Blood Serum**

**Submitting Doctor: Dr. Wang Xueqi  Clinical Diagnosis: Headache**

### Protein Quantitative Analysis

| Item | Cerebrospinal Fluid (mg/L) | Serum (g/L) | ALB Index | Reference Range (ALB Index) | IgG Index | Reference Range (IgG Index) |
|---|---|---|---|---|---|---|
| ALB | 6060 | 47.6 | | <9.0 | | <0.70 |
| IgG | 1250 | 13.6 | 127.31 ↑ | <0.70 | 0.75 ↑ | |

### 24h IgG Synthesis Rate

| - | Result (mg/24h) | Reference Range (mg/24h) |
|---|---|---|
| Tourtelotte Formula | 260.92 ↑ | -9.90 ~ 3.30 |

### Oligoclonal Band Electrophoresis (OBE)

☑**Type I**: No Oligoclonal Bands (OB) detected in both CSF and serum.

☐**Type II**: OB detected only in CSF.

☐**Type III**: OB detected in CSF with OB count > serum.

☐**Type IV**: OB detected in both CSF and serum, but with different patterns.

☐**Type V**: Identical OB patterns detected in both CSF and serum.

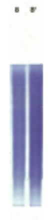

CSF S

寡克隆区带图谱

## Oligoclonal Band (OB) Electrophoresis Project Explanation

| Type | OB Interpretation | Implications | Clinical Significance |
|---|---|---|---|
| I | Negative | Central nervous system (CNS) has no intrathecal IgG synthesis. | Found in healthy individuals and patients with non-inflammatory neurological diseases (NIND). |
| II | Positive | OB detected in CSF due to CNS B-cell immune response, indicating intrathecal IgG synthesis. | Mainly observed in multiple sclerosis (MS) patients; occasionally seen in other CNS demyelinating diseases, autoimmune diseases, CNS infections, or rheumatic diseases. |
| III | Positive | OB detected in CSF with a pattern different from serum, due to systemic B-cell immune response affecting CSF. | Mainly seen in chronic inflammatory demyelinating polyneuropathy, meningitis, or encephalitis; occasionally in MS patients whose OB results may not fall into Type II. |
| IV | Negative | OB detected in both serum and CSF with identical patterns, suggesting systemic immune response outside CNS involvement. | Mainly found in systemic autoimmune diseases like lupus, some CNS demyelinating diseases other than MS, or CNS infections. |

| | | | |
|---|---|---|---|
| V | Negative | Suggests monoclonal gammopathy. | Mainly observed in blood disorders (e.g., multiple myeloma), some systemic inflammatory diseases, or lymphoma involving the CNS. |

**Note: Positive CSF oligoclonal bands (OB) indicate intrathecal synthesis, as seen in Type II and III, which assist in diagnosing multiple sclerosis (MS) but are not exclusive to MS.**

## Result Summary

**The cerebrospinal fluid (CSF) albumin index is elevated beyond the normal reference range. The 24-hour CSF IgG synthesis rate is also elevated beyond the normal reference range. No oligoclonal bands (OB) were detected in either CSF or serum. Please correlate with clinical findings for further analysis.**

# 涉及人的生物医学研究伦理审查批件

## Ethics Committee Approval Letter of Biomedical Research Involving Humans

批件号 **Approval NO.:** 浙人医伦审 **2023** 其他第（**407**）号

| | | | |
|---|---|---|---|
| 项目名称<br>**Study Title** | 基于脑脊液 IgG 指数和 24hIgG 合成率的联合检查对原发性中枢神经系统淋巴瘤（PCNSL-DLBCL）的诊断 | | |
| 申办方<br>**Sponsor** | 浙江省人民医院 | | |
| 受理号<br>**Acceptance Number** | QT2023408 | | |
| 主要研究者<br>**Principal Investigator** | 吴建国 | 承担科室<br>**Responsible Department** | 检验中心 |
| 审查类别<br>**Category of Review** | 初始审查 | 审查方式<br>**Type of Review** | 快速审查 |
| 审查日期<br>**Date of Review** | 2023 年 11 月 27 日 | 审查地点<br>**Location of Review** | 无 |
| 审查文件清单<br>**Items Reviewed** | 1.临床科研项目伦理审查申请表<br>2.研究方案（2023112101/2023-11-21）<br>3.临床试验豁免知情同意申请书<br>4.主要研究者简历 | | |
| 审评意见<br>**Evaluation** | 无 | | |
| 审查决定<br>**Decision** | 委员会对该项目的审查决定为： ☒同意（Approval） | | |
| 主任/副主任委员签字<br>**Chair Signature** | [signature] | | |
| 签发日期<br>**Date of issue** | 2023.11.28 | | |
| 伦理审查委员会<br>**Stamp of EC** | 伦理审查委员会盖章 | | |
| 批件有效期<br>**Period of Validity** | 自本伦理审查委员会初始审查批准之日起一年内，本临床研究应在本院启动。逾期未启动的，本批件自行废止。 | | |
| 年度/定期跟踪审查<br>**Continue Review** | 审查频率为该研究批准之日起每 12 个月一次，首次请于 2024 年 11 月 26 日前 1 个月递交研究进展报告。<br>伦理审查委员会有根据实际进展情况改变跟踪审查频率的权利。 | | |
| 声明<br>**Statement** | 本伦理审查委员会的职责、人员组成、操作程序及记录遵循《涉及人的生物医学研究伦理审查办法》、《涉及人的健康相关研究国际伦理准则》、《赫尔辛基宣言》、GCP 和 ICH-GCP 等国际伦理指南和国内相关法律法规。 | | |

注意事项：

1. 请遵循我国相关法律、法规和规章中的伦理原则。
2. 请遵循经本伦理审查委员会批准的临床研究方案、知情同意书、招募材料等开展本研究，保护受试者的健康与权利。对研究方案、知情同意书和招募材料等的任何修改，均须得到本伦理审查委员会审查同意后方可实施。
3. 在本院发生的SAE/SUSAR以及研发期间安全性更新报告须按照NMPA/GCP最新要求及时递交本伦理审查委员会，国内外其它中心发生的SAE/SUSAR需定期汇总、评估后递交本伦理审查委员会。
4. 根据报告情况，本伦理审查委员会有权对其评估做出新的决定。
5. 自今日起，无论研究开始与否，请在跟踪审查日到期前1个月提交研究进展报告。
6. 申办方应当向组长单位伦理审查委员会提交中心研究进展报告汇总；当出现任何可能显著影响研究进行或增加受试者危险的情况时，请申请人及时向本伦理审查委员会提交书面报告。
7. 研究纳入了不符合纳入标准或符合排除标准的受试者，符合中止研究规定而未让受试者退出研究，给予错误治疗或剂量，给予方案禁止的合并用药等没有遵从方案开展研究的情况；或可能对受试者的权益或健康以及研究的科学性造成不良影响等违背GCP原则的情况，请申办方、监查员或研究者提交违背方案报告。
8. 申请人暂停或提前终止临床研究，请及时提交暂停或终止研究报告。
9. 完成临床研究，请申请人提交结题报告。
10. 凡涉及中国人类遗传资源采集标本、收集数据等研究项目，必须获得中国人类遗传资源管理办公室批准后方可在本中心开展研究。
11. 凡经本伦理审查委员会批准的研究项目在实施前，申请人应按相关规定在国家卫健委、药审中心等的临床研究登记备案信息系统平台登记研究项目相关信息。

# Ethics Committee Approval Letter of Biomedical Research Involving Humans

**Approval No**.: Zhejiang Medical Ethics 2023 Total No. (407)

**Study Title** ： Diagnosis of Primary Central Nervous System Lymphoma (PCNSL-DLBCL) Based on Brain Nerve Biomarkers IgG Index and 24hIgG Synthesis Rate

**Sponsor** ： Zhejiang Provincial People's Hospital

**Acceptance Number** ： QT2023408

**Principal Investigator** ： Wu Jianguo **Responsible Department** ： Inspection Center

**Category of Review** ： Initial Review **Type of Review** ： Expedited Review

**Date of Review** ： November 27, 2023 **Location of Review** ： None

**Items Reviewed**

1. **Clinical Research Project Ethical Review Application Form**
2. **Research Proposal (20231121/2023-11-21)**
3. **Clinical Trial Exemption Consent Form**
4. **Principal Investigator's Resume**

**Evaluation** ： None
**Decision** ： Approval
**Chair Signature**

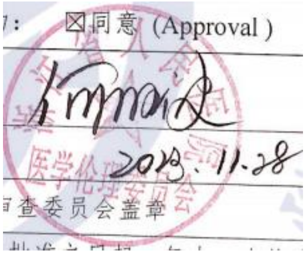


**Date of Issue :** November 28, 2023

**Stamp of EC:** Zhejiang Provincial Medical Ethics Committee
**Period of Validity** This ethical approval is valid for one year from the date of initial approval by the Ethics Committee. If the project is not initiated within this period, the approval will automatically become invalid.

**Continue Review** The initial review approval of the clinical research should be submitted for continuation review every 12 months, with the first submission before November 26, 2024.

**Statement** This ethical approval is based on the regulations, personnel requirements,

operational procedures, and environmental controls involved in biomedical research involving humans ("Management Measures for Ethical Review of Biomedical Research Involving Humans", "International Ethical Guidelines for Biomedical Research Involving Humans", Helsinki Declaration), GCP, and ICH-GCP international guidelines and relevant laws and regulations.

Medicine

# Declarations Form

## Declarations

### Disclosure of interest

We declare no conflicts of interest.

### Data availability

The materials described in the manuscript, including all relevant raw data, will be freely available to any scientist wishing to use them for non-commercial purposes without breaching participant confidentiality.

### Funding

The current study received funds from the Zhejiang Province Medical and Health Science and Technology Project, China (no. 2022KY877) and the Jiashan Bureau of Science and Technology, China (no. 2023A60).

### Ethical approval

Ethical approval was not required.

### Pre-registered clinical trial number

Not applicable.

**Highlights：**

1. A novel auxiliary diagnostic method combining CSF IgG index, IgG synthesis rate, and immunophenotyping.

2. Provides effective diagnostic support when MRI and PET-CT results are inconclusive.

3. Offers a noninvasive alternative for patients unsuitable for invasive brain biopsy.

4. Enhances early detection and supports timely clinical decision-making for PCNS-LBCL.
